# Supplementary material for: A scoping review of the post-discharge care needs of babies requiring surgery in the first year of life
Source: PLOS Glob Public Health. 2023 Nov 22;3(11):e0002424. doi: 10.1371/journal.pgph.0002424 (PMC10664918; doi:10.1371/journal.pgph.0002424)
Supplement: S1 Table — (PDF) [file pgph.0002424.s002.pdf]

S1 Table: Search Strategy developed with University Librarian

PUBMED 18 October 2022 (2646 results)

#	Search term	Search terms
1	Congenital conditions	(("hernias, diaphragmatic, congenital"[MeSH Terms] OR "Esophageal Atresia"[MeSH Terms] OR "Gastroschisis"[MeSH Terms] OR "Anorectal Malformations"[MeSH Terms] OR "Hypoplastic Left Heart Syndrome"[MeSH Terms] OR "Pulmonary Atresia"[MeSH Terms] OR "Tetralogy of Fallot"[MeSH Terms] OR "Scimitar Syndrome"[MeSH Terms] OR "Transposition of Great Vessels"[MeSH Terms] OR "Tricuspid Atresia"[MeSH Terms] OR "Truncus Arteriosus"[MeSH Terms] OR "Hypoplastic Left Heart Syndrome"[Text Word] OR "left heart hypoplasia"[Text Word] OR "HLHS"[Text Word] OR "congenital diaphragmatic defect*"[Text Word] OR "morgagni hernia*"[Text Word] OR "pulmonary valve atresia"[Text Word] OR "fallot tetralogy"[Text Word] OR "total anomalous pulmonary venous return"[Text Word] OR "scimitar anomal*"[Text Word] OR "pulmonary venous return anomaly"[Text Word] OR "TAPVR1"[Text Word] OR "TAPVR"[Text Word] OR "great vessels transposition"[Text Word] OR "dextro looped transposition of the great arteries"[Text Word] OR "absent right atrioventricular connection"[Text Word] OR "tricuspid valve atresia*"[Text Word] OR "bochdalek hernia*"[Text Word] OR "oesophageal atresia*"[Text Word] OR "gastroschises"[Text Word] OR "anorectal malformation*"[Text Word] OR "anorectal anomal*"[Text Word] OR "anorectal atresia*"[Text Word] OR "anorectal stenosis"[Text Word] OR "anorectal stenoses"[Text Word] OR "critical congenital heart defect*"[Text Word] OR "critical congenital heart disease*"[Text Word])
2	Home care/caregivers	("Patient Discharge"[MeSH Terms] OR "Aftercare"[MeSH Terms:noexp] OR "Caregivers"[MeSH Terms] OR "Parents"[MeSH Terms] OR "discharge*"[Text Word] OR "handover*"[Text Word] OR "hand over*"[Text Word] OR "handoff*"[Text Word] OR "hand off*"[Text Word] OR "transition"[Text Word] OR "transfer"[Text Word] OR "continuity of patient care"[Text Word] OR "home care"[Text Word] OR "after care"[Text Word] OR "after treatment"[Text Word] OR "follow-up care"[Text Word] OR "Aftercare"[Text Word] OR "caregiver*"[Text Word] OR "care giver*"[Text Word] OR "family*"[Text Word] OR "families*"[Text Word] OR "parent*"[Text Word] OR "stepparent*"[Text Word] OR "step parent*"[Text Word] OR "mother*"[Text Word] OR "father*"[Text Word] OR "mom"[Text Word] OR "dad"[Text Word])
3	Neonates	("Infant"[MeSH Terms] OR "neonat*"[Text Word] OR "newborn*"[Text Word] OR "new born*"[Text Word] OR "infant*"[Text Word] OR "baby*"[Text Word] OR "babies*"[Text Word] OR "child*"[Text Word]))
4	1 AND 2 AND 3	

Filtered to English and between 2002-2022 Search terms were adapted for EMBASE, CINAHL & PsychInfo databases.
