# Supplementary material for: A scoping review of the post-discharge care needs of babies requiring surgery in the first year of life
Source: PLOS Glob Public Health. 2023 Nov 22;3(11):e0002424. doi: 10.1371/journal.pgph.0002424 (PMC10664918; doi:10.1371/journal.pgph.0002424)
Supplement: S2 Table — Please note references [45, 54–68] are included in the scoping review, further information on these papers is included in the data extraction table. (PDF) [file pgph.0002424.s003.pdf]

S2 Table: DATA EXTRACTION TABLE

Studies of Cardiac conditions (n = 15)

First Author<br>Date<br>Location	Type of paper/study & Methods	Purpose	Condition/disease	Population	Complex need identified	Relevant Results
1.<br>Tregay et al.<br>2016<br>UK (1)	Qualitative<br>– Semi-structured interviews in-person in the caregivers' home (except one)<br>– Framework analysis	"To understand more about parent experiences of caring for a child with complex needs after congenital heart surgery"	CHD (HLHS[1], TGA[2], TOF[3], TAPVR[4])	21 caregivers who had a child with congenital heart surgery before the age of 1 year old between September 2009 and October 2013. Infants who required emergency readmission or died after discharge were the primary focus. The sample was taken from 3 UK cardiac centres; half of the infants were discharged on a form of assistive feeding device<br>(9 with NGT[5], 1 with gastrostomy)	– Feeding<br>– Oral intake monitoring<br>– Weight monitoring<br>– Managing assistive feeding devices[6]	80% of caregivers reported feeding difficulties. This included infant pulling out their NGT, taking a long time to feed, having to add several different medications to feeds and vomiting. Parents had to calculate amounts of feeds and monitor fluid intake and weight gain with charts.
2.<br>Oster et al.<br>2015<br>USA (2)	Retrospective review<br>– Data prospectively collected from the National Pediatric Cardiology Quality Improvement Collaboration<br>– Interstage mortality and unplanned readmissions compared with home oxygen saturation and	To determine the outcomes of neonates with HLHS who receive regular interstage home monitoring	HLHS	Infants with univentricular heart requiring Norwood procedure between 2008 to 2012. 494 participants included for home oxygen saturation monitoring and 472 for home weight monitoring	– Oxygen saturation monitoring<br>– Weight monitoring	Daily oxygen saturation monitoring, weekly monitoring and no monitoring was done for 80%, 12% and 7% of subjects respectively. For home weight monitoring, 75% were monitored daily, 13% weekly and 12% never. Those with daily weight monitoring had better interstage weight gain than those with no monitoring (ΔWAZ: −0.15±0.18; p <0.01). The major finding was that the time to stage II palliation operation was longer for those with no oxygen saturation or weight monitoring. But mortality and readmission rates did not differ significantly.

[1] HLHS, Hyperplastic left heart syndrome
[2] TGA, Transposition of the great arteries
[3] TOF, Tetralogy of Fallot
[4] TAPVR, Total anomalous pulmonary venous return
[5] NGT, Nasogastric tube
[6] Assistive feeding devices, Devices used to give feeds other than by mouth (e.g., nasogastric tube, gastrostomy tube etc.)

	weight monitoring					
3.<br>Imperial-Perez et al.<br>2021<br>USA (3)	Qualitative<br>– Data collection done between November 2019 to July 2020<br>– Sociodemographic questionnaires<br>– In-depth interviews (in-person/telephonic)<br>– Grounded theory analysis	To explore the experiences and perceptions of caregivers while looking after children in the interstage period	Single ventricle pathology (HLHS)	14 caregivers of infants with single-ventricle heart disease who had first-stage surgery and had been discharged for 2-4 months	– Oxygen saturation monitoring<br>– Weight monitoring<br>– Oral intake monitoring<br>– Extra attention/supervision	Caregivers reported being overwhelmed with care-giving responsibilities. Feeding was identified as a major challenge, including the fear of keeping their child hydrated and gaining weight adequately. "The intense feeding schedule led to exhaustion." Caregivers were instructed to measure oxygen saturation 2 – 3 times per day. 3-hourly tube feeding was also a requirement for some parents.
4.<br>Natarajan et al.<br>2009<br>USA (4)	Retrospective cohort study<br>– Review of medical records<br>– Statistical analyses to determine association of risk factors and feeding morbidity	To assess feeding patterns and risk factors for feeding-related morbidities in neonates with CHD[7]	CHD (HLHS, TAPVR, TOF, TGA, hypoplastic right heart, truncus arteriosus, tricuspid atresia, Ebstein's anomaly)	67 infants <1 month of age who underwent surgery for CHD between January 2002 and December 2008, at the Children's Hospital of Michigan	– Managing assistive feeding device	53.8% of infants discharged on full oral feeds and 40.3% with a feeding tube. 70% require enriched feeds at discharge. In those with HLHS, 45.2% died or had gavage at discharge, and 13.9% were discharged on full oral feeds. Feeding morbidities were 58.6% and 36.8% in univentricular vs biventricular physiology (statistically significant). The need for cardiopulmonary bypass and older age at full feeds prior to surgery were significantly related to death/gavage at discharge (p 0.024 and 0.039).
5.<br>Hill et al.<br>2014<br>USA (5)	Cross-sectional study<br>– Questionnaire (Mealtime Behaviour Questionnaire, About Your Child's Eating, and/or the Parenting Stress Index) administered to parents of patients presenting	To discover whether patients with single ventricle pathologies have more feeding difficulties compared to normal patients and	Single ventricle CHD (HLHS (n=4), pulmonary atresia (n=2), unbalanced atrioventricular septal defect (n=1), tricuspid atresia (n=1))	8 infants (mean age 36 months +/- 23 months) with single ventricle pathology	– Managing assistive feeding devices<br>– Feeding	8 patients (89%) had GT[8]. Choking, gagging, or vomiting was reported in all patients. Single ventricle patients had significantly more resistance to eating than normal controls (83 vs. 2.3% $p < 0.001$) and compared to noncardiac feeding disorder group (44%, $p = 0.05$).

[7] CHD, Congenital heart disease
[8] Gastrostomy tube

	to the Feeding, Swallowing, and Nutritional Center at the Children's Hospital of Wisconsin	those with noncardiac abnormalities				
6.<br>Siehr et al.<br>2014<br>USA (6)	Retrospective review<br>– Retrospective review of clinical data of participants at discharge and follow-up	"To present the results of 5 years of interstage home monitoring in modified Norwood procedure patients at the Lucile Packard Children's Hospital"	Single ventricle pathology (HLHS (n=37), critical aortic stenosis (n=1), AVSD[9] (n=3), double-outlet right ventricle/mitral atresia/hypoplastic arch (n=2), tricuspid atresia (n=2), double-inlet left ventricle (n=1))	46 patients with HLHS or single-ventricle lesions requiring Norwood procedure and who survived to discharge between October 2005 – 2010	– Oxygen saturation monitoring<br>– Weight monitoring<br>– Daily oral intake<br>– Managing assistive feeding devices<br>– Extra attention/supervision	Daily oxygen saturation, weight and oral intake monitoring required. Caregivers advised on red flags. 41% of patients had readmission during interstage period. All patients survived to second stage Glenn surgery. 37% of patients required NGT feeding and 17% required GT.
7.<br>Ghanayem<br>2002<br>USA (7)	Prospective cohort study<br>– Control group compared to intervention group (who received scales and pulse oximeters and advised to monitor weight and saturation)<br>– Clinical data collected retrospectively from medical records before discharge and	To determine whether home surveillance program reduces mortality in the interstage period	Single ventricle pathology (HLHS and HLHS with variants)	87 patients who underwent stage 1 palliative surgery for HLHS or a variant of HLHS at Children's Hospital of Wisconsin from July 1996 to November 2001	– Oxygen saturation monitoring<br>– Weight monitoring<br>– Daily oral intake<br>– Managing assistive feeding devices<br>– Extra attention/supervision<br>– Provision of supplemental oxygen	15.8% mortality in control group vs 0% in intervention group ($p = 0.039$). Age to stage 2 palliation surgery younger in intervention group (4.3 ± 1.6 months compared with 5.6 ± 2.1 months in control group ($p < 0.016$). 17.5% in intervention group and 16.7% in control group were discharged on supplemental oxygen. 28% and 25% of patients in intervention and control groups were discharged on GT.

[9] Atrioventricular septal defect

	prospectively at follow-up					
8.<br>Wray et al.<br>2018<br>UK (8)	Qualitative<br>– Semi-structured interviews (in-person and telephonic)<br>– Framework analysis	To discovery perceptions of helpline staff who consult telephonically with caregivers of infants discharged post-cardiac surgery	CHD	10 helpline staff from various charities associated with the Children's Heart Federation	– Feeding<br>– Managing assistive feeding devices<br>– Extra attention/supervision	Feeding was identified as a common reason for parents calling the helpline. This included managing tube feeding. Parents often called about looking out for signs and symptoms that were worrying (e.g., cyanosis). Parents were identified as being highly vigilant and anxious.
9.<br>Gaskin et al.<br>2016<br>UK (9)	Mixed methods study<br>– Semi-structured interviews (8 participants)<br>– The results of this interview used to develop an online survey that was completed by parents (22 participants)	To discuss the readiness and experience of parents after going home with infants post first-stage surgery for functionally univentricular heart	Single ventricle pathology (HLHS (n=14), hypoplastic right heart syndrome (n=8), tricuspid atresia, pulmonary atresia, unbalanced forms of transposition with small right ventricle)	30 parents (members of CHD charity) of infants who had undergone surgery for univentricular heart	– Feeding<br>– Extra attention/supervision	Parents felt unprepared either physically, emotionally, or educationally for their discharge home with their infant following the first stage of cardiac surgery. Parents reported fear of recognising when the child had decompensated and knowing exactly how to feed and monitor weight. A chart was developed to help parents categorise signs and symptoms according to urgency and act accordingly. Signs and symptoms include baby's skin colour, activity, breathing, circulation, feeding.
10.<br>Alten et al.<br>2015<br>North America (10)	Retrospective review<br>– Review of clinical data from the Pediatric Cardiac Critical Care Consortium (PC[4]) which has data from 21 North American centres	To describe perioperative feeding practices in children with CHD	CHD (truncus arteriosus (n=8), interrupted aortic arch (n=10), HLHS (n=43), coarctation of aorta (n=45), pulmonary atresia (n=13), TGA (n=55), other (n=77)).	251 neonates who were admitted to the ICU[10] from 1 October 2013 to 2 July 2014, who received surgery for CHD and were less than 28 days at the time of surgery	– Managing assistive feeding devices	43.8% of all patients were discharged with a feeding tube. 15.9% had surgical feeding tubes (GT/enterostomy) at discharge. Most patients with feeding tubes on discharge had stage I palliation for HLHS (56.9% with feeding tube and 22.4% with surgical feeding tube). In three centres no infants were discharged on surgical tubes vs. at three other centres where >75% had surgical feeding tubes.

[10] ICU, Intensive care unit

11.<br>Öhman et al.<br>2012<br>Sweden (11)	Prospective interventional study<br>– Rate of positive home monitoring recorded (successful admission because of saturation <70%)<br>– Compared with interstage mortality in patients from August 2002 – June 2007<br>– Questionnaire sent to parents in January 2010 in infants who completed stage II palliation or died	To assess whether daily oxygen saturation monitoring can help early detection of shunt occlusion, and parents' experiences of interstage monitoring.	Single-ventricle pathology (HLHS and variants)	28 infants with single-ventricle pathology who were operated on between September 2007 – December 2010 at Queen Silvia Children's Hospital in Gothenburg	– Oxygen saturation monitoring	Once daily oxygen saturation measurements done by parents. Sensitivity and specificity of home monitoring program was 63% and 90%. Positive predictive value = 71% and negative predictive value = 86%. Parents viewed oxygen saturation monitoring positively and none thought it was too much medical responsibility. 9/14 reported being worried when doing measurements.
12.<br>Tregay<br>2014<br>UK (12)	Qualitative<br>– Semi-structured interviews (in person)<br>– Framework analysis	To understand the perspectives of parents taking care of neonates with complex needs post congenital cardiac surgery	CHD (single-ventricle disease, HLHS, TGA, ventriculoseptal defect, TOF, anomalous coronary artery from pulmonary artery, TAPVR)	20 parents whose children were operated on for CHD (between September 2009 and October 2013) and subsequently died or had an unplanned admission to ICU following initial discharge	– Extra attention/supervision	Changes in feeding, gastrointestinal symptoms (vomiting), breathing, appearance (cyanosis) and behaviour changes were most common before deterioration. This highlights the need for extra attention/supervision. Parents were expected to monitor for signs and symptoms constantly.
13.<br>March et al.<br>2018<br>USA (13)	Qualitative<br>– Qualitative description of blog entries<br>– Thematic analysis	To understand the parents' experiences during transition from hospital to home-care in children with HLHS	HLHS	6 parents of children (from any country) with HLHS who wrote blogs regarding their experiences between November 2008 and May 2013	– Oxygen saturation monitoring<br>– Weight monitoring<br>– Daily oral intake<br>– Managing assistive	Parents reported being hyper-vigilant for signs suggesting decompensation. "I stayed up and watched her sleep the whole night." Many parents also took measures to protect their child from infection (not leaving home or allowing visitors). The use of NGTs and feeding was also highlighted as a challenge.

					feeding devices<br>– Extra attention/supervision<br>– Protecting from infection	
14.<br>Hartman et al.<br>2013<br>USA (14)	Qualitative<br>– Focus group discussions (for 15 caregivers)<br>– In-depth interviews (telephonic, for 4 caregivers)	To explore primary caregivers' perceptions of infant care post-discharge from surgery for CHD	CHD	19 caregivers (English speaking) of infants who had neonatal heart surgery for complex CHD and were discharged home between January 1 and June 30 2008	– Feeding<br>– Managing assistive feeding devices<br>– Extra attention/supervision	All caregivers attempted to feed every 2.5 to 3 hours. Feeding was challenging as infants "give up," take long to finish feeds or don't signal when hungry. Many found managing the NGT stressful and time-consuming and were concerned about whether they would be able to re-insert the tube if it dislodged. Ensuring enough caloric intake was a challenge, especially for children that vomit post-feeds. Many parents reported being hyper-vigilant for decompensation.
15.<br>Hansen et al.<br>2011<br>Germany (15)	Retrospective cohort study<br>– Analysed medical records of patients	To institute a home surveillance program for interstage monitoring	HLHS	45 patients who had home surveillance (between October 2005 – September 2009), 97 patients who had surgery before home monitoring implemented (before October 2005) and<br>20 infants not discharged after Norwood and before 2nd procedure	– Oxygen saturation monitoring<br>– Weight monitoring<br>– Daily oral intake<br>– Managing assistive feeding devices<br>– Extra attention/supervision<br>– Provision of supplemental oxygen	Daily oxygen saturation, fluid intake and weight monitoring done by caregivers in surveillance group. 25% of cases were discharged on feeding tube and 17% on supplemental oxygen. Post-home monitoring program implementation interstage mortality decreased from 12.4 to 2.2%.

Non-cardiac conditions (n = 25)

Author<br>Date<br>Location	Type of paper/study & Methods	Purpose	Condition/disease	Population	Complex need identified	Relevant results
1.<br>Anyanwu et al.<br>2013<br>Nigeria (16)	Descriptive study<br>– The study was conducted between January and December 2011<br>– Unstructured interviews conducted at first post-operative follow-up visit	To describe the improvised methods for stoma care used by parents	ARM[11], HD[12], anorectal injury	Mothers of 44 children who have colostomies (aged 1 day – 12 years)	– Stoma care	72.73% of participants had colostomy for ARM. 41.51% of mothers used diapers to collect effluent and 35.85% used a wrap-around waist band. Mothers were expected to clean around the stoma site and lubricate with petroleum jelly.<br>Majority of mothers struggled to follow stoma care instructions with most infants having varying degrees of skin excoriation.
2.<br>Ahmad et al.<br>2021<br>USA (17)	Prospective randomised control trial<br>– Patients were randomised to a dilatation or non-dilatation group<br>– Patients were observed for stricture formation or strictures requiring operative repair	To determine whether children having routine anal dilatations post-PSARP[13] have lower rates of strictures postoperatively	ARM	50 patients (median age 5 months) with ARM who had a primary PSARP (between March 2017 and September 2019) and were aged 24 months or younger	– Anal dilatations<br>– Stoma care	Two weeks post-surgery parents in dilatation group had to perform dilatations with Hegar dilators twice a day. 21% in dilatation group vs 32% in non-dilatation group developed strictures (p = 0.21).<br>Post-operative dilatation by parents does not significantly reduce stricture formation.<br>Colostomies were present in 71% and 52% of patients in the dilatation and non-dilatation groups respectively.
3.<br>Marten et al.<br>2011	Cross-sectional study<br>– Caregivers rated HRQoL on EQ-	To discuss the caretaking activities for	ARM and CDH[16]	164 parent-caregivers of children (1-11 years old) born with ARM or CDH who had received neonatal surgery	ARM<br>– Stoma care	48% of mothers and 32% of fathers of children with ARM believed that caretaking duties were more time

[11] ARM, Anorectal malformation
[12] HD, Hirschsprung's disease
[13] PSARP, Posterior sagittal anorectoplasty
[16] CDH, Congenital diaphragmatic hernia

The Netherlands (18)	VAS[14] questionnaire and then rated HRQoL under the hypothetical that someone would take over their caregiving duties	caregivers of children with major congenital abnormalities and how this affects their HRQoL[15].			– Giving enemas<br>– Changing diapers/washing more often<br>– Extra attention/supervision<br>CDH<br>– Provision of supplemental oxygen<br>– Feeding requirements<br>– Extra attention/supervision<br>– Administering medication<br>– Visiting health care providers	consuming than that for children without congenital abnormalities of the same age. For CDH this was 13%. 20% of parents of children with ARM and 44% of those with CDH described having to provide supervision/extra attention in general/cheering up. 25% of parents of children with CDH mentioned having to prepare special meals/help with eating.
4.<br>Zenilman et al.<br>2022<br>USA (19)	Retrospective review<br>– Retrospective analysis of data from DHREAMs study (demographic data, gestational age and weight, outcomes)	To characterise risk factors in neonates with CDH who are small for gestational age	CDH	584 neonates enrolled in the DHREAMS (Diaphragmatic Hernia Research and Exploration, Advancing Molecular Science) study from 12 centres (between 2005-2019)	– Provision of supplemental oxygen	15.3% of the neonates were SGA. Overall mortality higher in SGA (35.6%) vs AGA (19.4%) (p = 0.0007). Need for oxygen at 28 days 77.4% in SGA vs 55.6% in AGA (p – value = 0.003). Need for oxygen at discharge in SGA (19.3%) vs AGA (15.5%) (p = 0.46).
5.<br>Vu et al.<br>2017<br>USA (20)	Retrospective review<br>– Demographic data collected	To determine the nutritional outcomes and risk factors for	CDH	67 patients who had repair of CDH as neonates between 2000 – 2014 at University of California San Francisco Benioff Children's Hospital	– Feeding<br>– Managing assistive	13% of patients were discharged with a feeding tube and 6% with fortified feeds. At 6-month follow-up, 15% of patients required a feeding tube and 69% fortified

[14] EQ-VAS, Questionnaire that measures patients' self-reported health status on a visual analogue scale from 0 (worst imaginable health) to 100 (best imaginable health)
[15] HRQoL, Health-related quality of life

	from medical records<br>– Outcomes (weight, length, tube feeding, GT placement) were recorded at discharge, 6 months, and 12 months	poor outcomes of patients post-primary repair of CDH			feeding devices	feeds. 4 patients (median age 1.9 months) required GT.
6.<br>Osifo et al.<br>2008<br>Nigeria (21)	Retrospective review<br>– Records of patients receiving colostomy reviewed and data including the age at colostomy, indications and complications were recorded	To determine the main indications for colostomy and stoma-related complications	ARM, HD	46 patients who had colostomy performed at the University of Benin Teaching Hospital between January 1997 to December 2006	– Stoma care	48% of patients requiring stoma were for ARM. Morbidity and mortality higher for ARM than HD (P = 0.0021). Mean duration of stoma was 9.1 months (± 2.12). Poor parental acceptance was recorded in 21.7% of cases. Other complications were skin excoriation (84.8%), prolapse (32.6%), haemorrhage (28.3%) and sepsis/wound infection (26.1%).
7.<br>Ameh et al.<br>2006<br>Nigeria (22)	Prospective cohort study<br>– A simple structured questionnaire completed by caregivers attending outpatient clinic	To determine the acceptability of colostomy by parents in developing countries	ARM, HD	57 caregivers of children with colostomies completed questionnaires between January 1999 and January 2004	– Stoma care	28 of the 57 children had stomas for ARM 23% found the colostomy “unacceptable.” The main reason for this was social isolation. Other problems with stoma care included disturbing smell (30%), frequent changing of cloth fashioned as stoma bag (at least 5 times a day) (26%)
8.<br>Putnam et al.<br>2016<br>USA (23)	Prospective cohort study<br>– Data collected from the CDH Study Group (CDHSG) – an inventory of CDH data from over 50 hospitals	To determine common morbidities associated with CDH and the characteristics that predict discharge morbidity	CDH	3665 patients from 60 centres in 13 countries	– Provision of supplement al oxygen<br>– Managing assistive feedings devices	19.2% of patients were discharged on supplemental oxygen. 30.5% were discharged with supplemental tube feeds. Defect size was the greatest predictor of overall morbidity (OR 11.23 (4.81-26.24) P <0.001).
9.<br>Emil et al.<br>2011	Retrospective review<br>– Review of patient records	To review the outcomes and patient	Gastroschisis	83 patients treated for gastroschisis at the University of California Irvine Medical	– Managing assistive	1 in 4 patients had complex gastroschisis (gastroschisis with 1 or more of the following associated complications:

USA (24)		progress from complex gastroschisis over a 6-year period		Center between January 2001 and March 2007	feeding devices	intestinal atresia, stenosis, volvulus, perforation, gangrene, or closing gastroschisis). 1/3 of patients were discharged on oral feeding only, 1/3 on oral and GT feeding (without TPN[17]) and 1/3 on combination of enteral and parenteral nutrition.
10.<br>Chu and Duong 2015<br>Vietnam (25)	Mixed methods study<br>– Outcomes of intervention group (group receiving education) vs. control group (no education) measured<br>– Semi-structured interviews at delivery, after stoma surgery, before discharge, one month post discharge<br>– Content analysis	To evaluate an educational program developed to assist parents of children with ARM	ARM	20 parents of newborns with ARM awaiting colostomy procedure at the surgical department of the National Hospital of Pediatrics, Hanoi	– Stoma care<br>– Feeding	Parents who received education were more confident with stoma care. Having to deal with effluent from stomas and complications at home was an issue. Stoma bags were unaffordable for most. Weight of infants in intervention group was significantly higher than control group after 1 month (weight gain 1.09kg (0.1-1.3) vs 0.067 (0.4 – 1.1) $p \leq 0.001$. Those in the intervention group also had fewer complications than control $p \leq 0.05$.
11.<br>Robatmily et al. 2018<br>Iran (26)	Clinical trial<br>– Mothers randomly assigned to control or intervention group (stoma education)<br>– Demographic questionnaire<br>– Skin examination checklist completed at discharge and 1 month after	"To [evaluate] the effect of providing ostomy care education to mothers of infants with peristomal skin complications."	ARM	40 mothers of neonates with ARM that required intestinal stomas from 3 institutions (Mofid, Ali Asghar, and Bahrami).	– Stoma care	There was a significant difference in skin condition in the two groups: 30% of infants in control group had healthy skin vs 80% in the intervention group ($p = 0.013$).

[17] TPN, Total parenteral nutrition

12.<br>Shwab et al.<br>2021<br>USA (27)	Retrospective review<br>– Review of patient charts between 2012 and 2020 at UCSF Benioff Children's Hospital	To identify the factors that increase GT requirement in infants born with CDH and evaluate the long-term growth of patients with GT	CDH	101 infants born with CDH who underwent surgical repair at UCSF Benioff Children's Hospital	– Managing assistive feeding devices	37.6% of neonates required a GT for nutritional support. 23.7% of these patients still had GT in situ by study completion. Tube-fed CDH infants were at higher risk of weight loss (35% dropped to a weight below the 5th percentile).
13.<br>Najaf et al.<br>2013<br>USA (28)	Retrospective review<br>– Retrospective data collection from medical records	To review morbidity and follow-up in survivors of CDH to help develop a multidisciplinary follow-up program	CDH	26 infant survivors born with CDH at St. Louis Children's Hospital from January 1 2006 – December 31 2010	– Managing assistive feeding devices<br>– Provision of supplemental oxygen	3 patients (11%) were discharged on supplemental oxygen and 9 (34%) needed supplemental tube feeds. 4 patients (18%) were still on tube feeds at follow-up. By 24 months three patients were weaned off supplemental oxygen.
14.<br>Baroudi et al.<br>2021<br>USA (29)	Cross sectional study and retrospective review<br>– Review of medical charts<br>– Questionnaires completed by parents on infant symptoms and parent QoL (The PedsQL Family Impact Module[18])	To assess the HRQoL for caregivers of infants with CDH and the risk factors for worse HRQoL	CDH	29 caregivers of infants with CDH presenting to Johns Hopkins Children's Center between June 2017 and April 2019	– Managing assistive feeding devices<br>– Provision of supplemental oxygen	15.5% of patients required home respiratory support. This was associated with lower HRQoL scores (p 0.041). 58.6% of patients required a feeding tube. Mean QoL low compared to other congenital respiratory diseases.
15.<br>Temple et al.<br>2011<br>Canada (30)	Retrospective review<br>– Review of surgical records<br>– Daily dilatation compared to those receiving weekly calibration	To determine whether daily anal dilatation is necessary for improved outcomes after surgery for ARM	ARM and HD	61 children with ARM and 34 with HD who had pull-through procedures between January 2005 and 2010 at the Hospital for Sick Children, Toronto	– Anal dilatation	Parents expected to perform daily or twice daily finger/Hegar anal dilatations. There was no statistically significant difference in complication rates in group with daily dilatation vs weekly calibration. 2% in daily dilatation vs 8% in weekly calibration required surgery for late narrowing (p = 0.38.

[18] PedsQL Family Impact Module, An instrument developed to measure the quality of life of parents and families of children with chronic medical conditions(41)

16.<br>Lees et al.<br>2018<br>Canada (31)	Retrospective cohort study<br>– Retrospective analysis of medical records	To explore the feeding outcomes in infants born with type-C oesophageal atresia and tracheoesophageal fistula	OA/TOF[19]	57 infants undergoing surgery for type-C OA/TOF repair at the University of Alberta Hospital in Edmonton, Canada between January 2005 – December 2015	– Managing assistive feeding devices	61.4% of patients were discharged on full oral feeds, 17.5% a combination of NGT and PO[20], 10.5% on NGT only, 1.8% on NGT and parenteral nutrition (PN), and 1.8% on NG, PO and PN.
17.<br>Akshaya et al.<br>2007<br>USA (32)	Retrospective review<br>– Retrospective analysis of medical records	To measure the outcome of patients admitted with gastroschisis to improve counselling of families	Gastroschisis	70 infants born with gastroschisis at the St. Louis Children's Hospital between 2000 – 2005	– Feeding<br>– Managing assistive feeding devices	3 infants were discharged on TPN. 14 infants were discharged on GT feeds (14% had simple gastroschisis and 50% had complex gastroschisis).
18.<br>Jenetzky<br>2012<br>Germany (33)	Cross-sectional study<br>– Participants interviewed with standard case-report forms<br>– Questionnaires relating to practice of anal dilatation completed by parents	To describe features of anal dilatation and risk factors for increased painful dilatation	ARM	346 patients (aged 1-12 years) with ARM	– Anal dilatations	88% of patients received dilatations for a median of 7 months (1-156 months). 81% received daily home dilatations of these 51% were dilated once a day and 30% at least twice daily. By the end of the treatment regime 32% had weekly dilatations, 20% every second day, and 43% still once or twice a day. 69% reported at least one painful dilatation with 43% of these rating pain intensity as "High." No significant difference in stricture rate in those dilated vs not (p = 0.775).
19.<br>Pierog et al.<br>2014<br>USA (34)	Retrospective review<br>– Review of electronic medical records<br>– Data also collected from DHREAMS study	To identify predictors for low weight and tube-dependent feeding at 1 year of age of infants with CDH	CDH	69 infants who were admitted in to NICU[21] with CDH between January 1 2007 and June 30 2012	– Managing assistive feeding devices	13 patients (18.8%) required tube feeding at 1 years old. Reasons were inability to take enough nutrition orally, or wean off tube due to oral aversion or feeding intolerance. Of these patients 54% were on GT, 15% on GT/fundoplication, 15% with jejunostomy and 15% with gastrojejunostomy.

[19] OA/TOF, Oesophageal atresia/tracheoesophageal fistula
[20] PO, Per os (by mouth)
[21] NICU, Neonatal intensive care unit

20.<br>Wong et al.<br>2019<br>Canada (35)	Retrospective review<br>– Retrospective review of medical records	Determine risk factors for tube feeding and long-term outcomes post tube-feeding in CDH patients	CDH	160 neonates with Bochdalek hernia treated from January 1 2000 to December 31 2013 at the Hospital for Sick Children Canada	– Managing assistive feeding devices	20% of patients required tube feeding at discharge. Patch repair, ECMO, initial arterial blood gas pH < 7.25, prolonged ICU stay, ventilator days, and days until first feed were associated with tube feeding at discharge ($p < 0.05$). 57.1% of fully tube fed and 81.8% of partially tube fed discontinued tube feeding in the long-term.
21.<br>Mullassery et al.<br>2018<br>UK (36)	Retrospective review<br>– Review of medical records<br>– Comparison of outcomes from two centres (centre A where twice daily dilatations routinely done and centre B where dilatation only done if evidence of stricture)	To determine whether routine anal dilatations reduce stricture and re-operation rates post PSARP	ARM	103 patients admitted for surgery for ARM between January 2011 and December 2015 at two different centres in the UK	– Anal dilatations	Twice daily dilatations done by parents at home. For those in centre A dilatations were considered for a median of 112 days (IQR 83-148 days). There was no statistically significant difference in redo procedures between centre A and B
22.<br>Krois et al.<br>2017<br>Honduras (37)	Cross-sectional study<br>– Questionnaire completed by families	To explore the impact of colostomy on families of children with stomas	ARM and HD	20 families of children (aged 1 week – 27 years) who had been treated with colostomy (18 for ARM and 2 for HD)	– Stoma care	One family (5%), 25% and 15% reported feeling disease always, often and sometimes had a negative impact on their lives respectively. 40%, 10% and 25% reported the stoma always, often, and sometimes negatively impaired their daily life respectively. Increased costs for care had a negative impact on 95% of families. Cost of stoma care was $86/month on average. Diapers were used in 55% to cover stoma and 45% used sheets. None used stoma bags. 85% did not experience any medical stoma complications.
23.<br>Gischler et al.<br>2008<br>The Netherlands (38)	Observational study<br>– Telephone calls of parents with congenital	To identify the reasons that parents of children with congenital	Congenital anomalies (OA (n=77), CDH (n=70), abdominal wall	468 parents of patients born with severe congenital anomalies treated by paediatric surgery department at Sophia Children's Hosptial between 2000 to 2006	– Feeding<br>– Managing assistive feeding devices	Most telephone calls during the period were for feeding issues (23.7%), followed by intestinal problems (diarrhoea, constipation 16.4%), respiratory problems (16%), fever (8.2%) and medication

	anomalies over 5 years analysed	anomalies use a telephonic helpline	defects (n=73), ARMs/HD (n=70), small intestinal anomalies (n=110), miscellaneous (n=68))			(6.9%). Calls about drains or tubes constituted 3.4%. Those requiring medical aids at discharge were more likely to call (p = 0.008), as were those with CDH and OE (p = 0.06 and 0.001).
24.<br>Jumbi et al.<br>2019<br>Kenya (39)	Descriptive cross-sectional study<br>– Data were collected from medical records<br>– Caregivers were asked closed-ended questions about anal dilatations	To explore the effectiveness of digital (finger) dilatation to prevent stricture formation	ARM	50 patients at Kenyatta Hospital with ARM awaiting colostomy closure	– Anal dilatations	46% of patients had anal dilatation done with adequately sized digits. Anal strictures occurred in 22% of patients, mostly due to non-compliance (73% reported inconsistencies with protocol). 32% had complications (painful dilatations, wound dehiscence, bleeding).
25.<br>Muzira et al.<br>2018<br>Uganda (40)	Mixed methods study (retrospective review with qualitative study)<br>– Review of the paediatric surgery operative log and medical records at<br>– Focus group discussions (n=20)<br>– Pilot survey developed from FGDs and administered in outpatient clinic (n=15)	To assess delays in care of children with ARM and HD. And assess the socioeconomic impact of surgical conditions requiring colostomy.	ARM (n=234), HD (n=114), gangrenous ileocolic intussusception (n=95), typhoid-induced intestinal perforation (n=50)	92 patients who were on the operative log for colostomy. 35 family members of patients with colostomy.	– Stoma care<br>– Anal dilatations<br>– Wound care	Many patients are still waiting for definitive correction of ARM and HD. No caregivers had access to stoma bags and most used cut up pieces of bedsheets to cover the stoma. Parents report that maintaining stoma hygiene is time consuming. Many participants had to leave work to take up extra caregiving duties. Wound care and anal dilatations were also brought up as difficult care responsibilities. Social stigma regarding the child's colostomy limited integration of families with their communities.

References:

1. Tregay J, Brown K, Crowe S, et al. “I was so worried about every drop of milk” – feeding problems at home are a significant concern for parents after major heart surgery in infancy. Matern Child Nutr. 2017 Apr 1;13(2).
2. Oster ME, Ehrlich A, King E, et al. Association of interstage home monitoring with mortality, readmissions, and weight gain: a multicenter study from the National Pediatric Cardiology Quality Improvement Collaborative. Circulation. 2015 Aug 11;132(6):502-8.
3. Imperial-Perez F, Heilemann MV, Doering LV, et al. Developing a sense of self-reliance: caregivers of infants with single-ventricle heart disease during the interstage period. Cardiol Young. 2022 Mar 24;32(3):465–71.
4. Natarajan G, Reddy Anne S, Aggarwal S. Enteral feeding of neonates with congenital heart disease. Neonatology. 2010 Nov;98(4):330–6.
5. Hill G, Silverman A, Noel R, et al. Feeding dysfunction in single ventricle patients with feeding disorder. Congenit Heart Dis. 2014 Jan;9(1):26–9.
6. Siehr SL, Norris JK, Bushnell JA, et al. Home monitoring program reduces interstage mortality after the modified Norwood procedure. Journal of Thoracic and Cardiovascular Surgery. 2014;147(2).
7. Ghanayem NS, Hoffman GM, Mussatto KA, et al. Home surveillance program prevents interstage mortality after the Norwood procedure. Journal of Thoracic and Cardiovascular Surgery. 2003;126(5):1367–75.
8. Wray J, Tregay J, Bull C, Knowles RL, et al. Issues facing families of infants discharged after cardiac surgery: the perceptions of charity helpline staff. Acta Paediatrica, International Journal of Paediatrics. 2018 Aug 1;107(8):1418–26.
9. Gaskin KL, Barron DJ, Daniels A. Parents’ preparedness for their infants’ discharge following first-stage cardiac surgery: Development of a parental early warning tool. Cardiol Young. 2016 Oct 1;26(7):1414–24.
10. Alten JA, Rhodes LA, Tabbutt S, et al. Perioperative feeding management of neonates with CHD: Analysis of the Pediatric Cardiac Critical Care Consortium (PC4) registry. Cardiol Young. 2015 Dec 1;25(8):1593–601.
11. Öhman A, Strömvall-Larsson E, Nilsson B, et al. Pulse oximetry home monitoring in infants with single-ventricle physiology and a surgical shunt as the only source of pulmonary blood flow. Cardiol Young. 2013 Feb;23(1):75–81.
12. Tregay J, Brown KL, Crowe S, et al. Signs of deterioration in infants discharged home following congenital heart surgery in the first year of life: A qualitative study. Arch Dis Child. 2016 Oct 1;101(10):902–8.
13. March S. Parents’ perceptions during the transition to home for their child with a congenital heart defect: How can we support families of children with hypoplastic left heart syndrome? Journal for Specialists in Pediatric Nursing. 2017 Jul 1;22(3).
14. Hartman DM, Medoff-Cooper B. Transition to home after neonatal surgery for congenital heart disease. MCN The American Journal of Maternal/Child Nursing. 2012 Mar;37(2):95–100.
15. Hansen JH, Furck AK, Petko C, et al. Use of surveillance criteria reduces interstage mortality after the Norwood operation for hypoplastic left heart syndrome. European Journal of Cardio-thoracic Surgery. 2012 May 1;41(5):1013–8.
16. Anyanwu LJ, Mohammad A, Oyebanji T. A descriptive study of commonly used postoperative approaches to pediatric stoma care in a developing country. Ostomy Wound Manage. 2013 Dec 1;59(12):32-7.
17. Ahmad H, Skeritt C, Halleran DR, et al. Are routine postoperative dilations necessary after primary posterior sagittal anorectoplasty? A randomized controlled trial. J Pediatr Surg. 2021 Aug 1;56(8):1449–53.
18. Poley MJ, Brouwer WBF, van Exel NJA, et al. Assessing health-related quality-of-life changes in informal caregivers: An evaluation in parents of children with major congenital anomalies. Quality of Life Research. 2012 Jun;21(5):849–61.
19. Zenilman A, Fan W, Hernan R, et al. Being small for gestational age is not an independent risk factor for mortality in neonates with congenital diaphragmatic hernia: a multicenter study. Journal of Perinatology. 2022 Sep 1;42(9):1183–8.
20. Vu LT, McFarland C, Bratton B, et al. Closer Look at the Nutritional Outcomes of Patients after Primary Repair of Congenital Diaphragmatic Hernia. J Pediatr Gastroenterol Nutr. 2017 Aug 1;65(2):237–41.
21. Osifo OD, Osaigbovo EO, Obeta EC. Colostomy in children: Indications and common problems in Benin city, Nigeria. Pak J Med Sci. 2008;24(2):199-203.
22. Ameh EA, Sabiu LK, Mshelbwala PM, et al. Colostomy in Children – an Evaluation of Acceptance among Mothers and Care-Givers in a Developing Country. South African journal of surgery. 2006;44(4).
23. Putnam LR, Harting MT, Tsao K, et al. Congenital diaphragmatic hernia defect size and infant morbidity at discharge. Pediatrics. 2016 Nov 1;138(5).
24. Emil S, Canvasser N, Chen T, et al. Contemporary 2-year outcomes of complex gastroschisis. J Pediatr Surg. 2012 Aug;47(8):1521–8.
25. Chu TH, Duong TH. Education of Parents When a Child Born with an Imperforate Anus; Does It Improve the Health of the Child? Open J Pediatr. 2015;05(01):90–8.
26. Robatmily A, Anboohi SZ, Shirinabadi Farahani A, et al. Effect of Providing Ostomy Care Education to Mothers of Neonates with Peristomal Skin Complications. 2018;27(4).
27. Schwab ME, Burke S, Klarich MK, et al. Factors and Growth Trends Associated With the Need for Gastrostomy Tube in Neonates With Congenital Diaphragmatic Hernia. J Pediatr Gastroenterol Nutr. 2021 Oct 1;73(4):555–9.
28. Najaf TA, Vachharajani AJ, Warner BW. Follow up of children with congenital diaphragmatic hernia and development of a multidisciplinary care program. online J Pediatr Neonatol. 2013;16.
29. Al Baroudi S, Collaco JM, Nies MK, et al. Health-related quality of life of caregivers of children with congenital diaphragmatic hernia. Pediatric pulmonology. 2021 Jun;56(6):1659-65.

30. Temple SJ, Shawyer A, Langer JC. Is daily dilatation by parents necessary after surgery for Hirschsprung disease and anorectal malformations? J Pediatr Surg. 2012 Jan;47(1):209–12.
31. Lees MC, Bratu I, Yaskina M, et al. Oral feeding outcomes in infants with esophageal atresia and tracheoesophageal fistula. J Pediatr Surg. 2018 May 1;53(5):929–32.
32. Vachharajani AJ, Dillon PA, Mathur AM. Outcomes in neonatal gastroschisis: An institutional experience. Am J Perinatol. 2007 Sep;24(8):461–5.
33. Jenetzky E, Reckin S, Schmiedeke E, et al. Practice of dilatation after surgical correction in anorectal malformations. Pediatr Surg Int. 2012 Nov;28(11):1095–9.
34. Pierog A, Aspelund G, Farkouh-Karoleski C, et al. Predictors of low weight and tube feedings in children with congenital diaphragmatic hernia at 1 year of age. J Pediatr Gastroenterol Nutr. 2014 Oct 8;59(4):527–30.
35. Wong MKW, Haliburton B, Graham A, et al. Requirement and Duration of Tube Feed Supplementation among Congenital Diaphragmatic Hernia Patients. J Pediatr Surg. 2019 May 1;54(5):895–8.
36. Mullassery D, Chhabra S, Babu AM, et al. Role of Routine Dilatations after Anorectal Reconstruction-Comparison of Two Tertiary Centers. European Journal of Pediatric Surgery. 2019 Feb 28;29(3):243–6.
37. Krois W, Dingemans AJM, Hernández PX, et al. Sociodemographics and the impact of a colostomy to indigent families and children with colorectal disorders in Honduras. J Pediatr Surg. 2018 Apr 1;53(4):841–6.
38. Gischler SJ, Mazer P, Poley MJ, et al. Telephone helpline for parents of children with congenital anomalies. J Adv Nurs. 2008 Dec;64(6):625–31.
39. Jumbi T, Kuria K, Osawa F, et al. The effectiveness of digital anal dilatation in preventing anal strictures after anorectal malformation repair. J Pediatr Surg. 2019 Oct 1;54(10):2178–81.
40. Muzira A, Kakembo N, Kisa P, et al. The socioeconomic impact of a pediatric ostomy in Uganda: a pilot study. Pediatr Surg Int. 2018 Apr 1;34(4):457–66.
41. Varni JW, Sherman SA, Burwinkle TM, et al. The PedsQL™ Family Impact Module: Preliminary reliability and validity. Health Qual Life Outcomes. 2004 Sep 27;2.
