# Supplementary material for: A scoping review of the post-discharge care needs of babies requiring surgery in the first year of life
Source: PLOS Glob Public Health. 2023 Nov 22;3(11):e0002424. doi: 10.1371/journal.pgph.0002424 (PMC10664918; doi:10.1371/journal.pgph.0002424)
Supplement: Attachment — Submitted filename: Response to reviewers.docx [file pgph.0002424.s004.docx]

**Response to reviewers**

Dear Reviewers

We would like to sincerely thank you for the detailed feedback and constructive comments on our manuscript titled “A scoping review of the post-discharge care needs of babies requiring surgery in the first year of life.” We appreciate the time and effort invested in reviewing our work, and we believe that the feedback has significantly improved the quality of our manuscript. Below, we have attached responses to each of your comments and have submitted a revised manuscript with track changes.

**Responses to Reviewer 1:**

- **Comment 1:** *Add "systematic review" to the title instead of scoping.*

**Response:**

Thank you for this comment. This review was informed by the

Joanna Briggs Institute’s methodological framework for scoping reviews, however, we	recognise that the paper has taken a systematic style and so in response we	have added, to the title, “a systematic scoping review.” Please kindly find tracked change in the title.

- **Comment 2:** *Consider starting with discussing Monitoring then Feeding. As vital signs reflect a more critical baby than feeding.*

**Response:**

Thank you for this comment. We agree this is more logical and have changed the order of care needs (discussing monitoring first then feeding) in the following sections (please see tracked changes highlighted in the revised manuscript):

a. Abstract
b. Table 3
c. Results
d. Discussion
e. Conclusion
- **Comment 3:** *Some conditions require special care were not mentioned as Cleft conditions.*

**Response:**

We thank you for this valuable insight. We considered this when designing this research but wanted to keep the scope to those conditions that, without treatment, would cause death within the first year of life and contribute to infant and under-five mortality. We have clarified this in our methods and understand this as a limitation and so have also added this to our limitations section.

- **Comment 4:** *Consider adding a discussion about multidisciplinary teams/roles from surgeons, counselors, nurses, and physiotherapists.*

**Response:**

Thank you for this suggestion. We agree that this is important and so have	included a section about the multidisciplinary team in the discussion.

**Responses to Reviewer 2:**

- **Comment 1:** *Language and inclusion of LMIC studies.*

**Response:**

We really value this comment and understand that this is a limitation of our search strategy. For practical purposes, given English is the first language of all authors, we only included English studies. While we recognise the value of having non-English papers for inclusive representation, we unfortunately do not currently have the resources to translate non-English papers. We acknowledge this as a major limitation of the study and have highlighted this in our limitations (see tracked changes), as well as reflected this in the sub-headings of the discussion and within the discussion points (see tracked changes). We recognise the importance of journals from non-English speaking areas. We have thus incorporated this feedback into our future work by engaging communities from different countries and languages to mitigate linguistic disparity and ensure valuable insights from the context of non-English speakers is included in future research.

- **Comment 2:** *Quality Assessment in Scoping Reviews:*

*While it's true that quality assessment is not a standard component of a scoping review, it's possible to do it. I would reframe the first sentence of the limitations section that affirms that quality assessment is not a part of scoping reviews.*

**Response:**

Thank you for this comment. We agree and have added this sentence in the	limitations section as advised (please see tracked changes).

- **Comment 3:** *Future directions:*

*I would like to know how the presented study can contribute to the following research agenda. What should be the focus of future studies based on your results? And I do not agree with the author's conclusion that further studies in LMICs are needed as other languages were not considered. Maybe, there are plenty of studies there.*

**Response:**
Thank you for the comment – it is much appreciated. This study will contribute to the future research agenda by considering those aspects of post-discharge care most important for families and caregivers of babies with complex care needs following discharge from hospital after surgery, to design interventions in this area. The review has identified important knowledge gaps in relation to the post-discharge care needs of surgical neonates and will form the basis of future in-depth contextual work in this area. Based on your feedback, we will also be designing work which includes a review of non-English publications and guidelines with our team. We have already taken the first steps to establish key contacts in these communities to ensure we have inclusive representation in the work that arises from this research. We have added more detail regarding this to the manuscript’s conclusion.

We are very grateful for the feedback and think that your comments have considerably strengthened our paper. We are planning a cohort study of these babies and have included countries and representations that are non-English speaking. We believe that with these additions and future studies, our research will significantly contribute to improving the post-discharge care needs of babies with complex care needs. We look forward to your feedback and thank you once again for your comments.

Yours sincerely,

Dr Francesca Maraschin, Dr Jackyln Adella and Dr Shobhana Nagraj

*Please note: all references have been updated according to changes*
