# Supplementary material for: A scoping review of the post-discharge care needs of babies requiring surgery in the first year of life
Source: PLOS Glob Public Health. 2023 Nov 22;3(11):e0002424. doi: 10.1371/journal.pgph.0002424 (PMC10664918; doi:10.1371/journal.pgph.0002424)
Supplement: Attachment — Submitted filename: Response to reviewers.docx [file pgph.0002424.s005.docx]

**Response to reviewers**

Dear Editor

We would like to sincerely thank you for the review and feedback on our manuscript titled “A scoping review of the post-discharge care needs of babies requiring surgery in the first year of life.” We appreciate the time and effort invested in reviewing our work, and we believe that the feedback has significantly improved the quality of our manuscript. Below, we have attached a response to the comment you made regarding the title of this paper.

**Response to The Editor:**

- **Comment 1:**
  “My only commentary is that this manuscript cannot be both a scoping review and a systematic review, so I take issue with the change that you have made to the title. Based on your methodology, it seems you have conducted a scoping review. If you believe otherwise, please justify this. If you agree that it is a scoping review, please make the change to the title and anywhere else in the manuscript where "systematic review" is mentioned.”

**Response:**

Thank you for this comment. We agree that the paper follows a scoping review methodology and have thus made the necessary changes to the title and text as suggested.

Thank you for this feedback and we look forward to hearing back from you.

Yours sincerely,

Dr Francesca Maraschin, Dr Jackyln Adella and Dr Shobhana Nagraj

*Please note: all references have been reviewed for retractions (of which there are none) and updated according to any manuscript changes*
